# Tandem Fluorescent Protein Timers for Noninvasive Relative Protein Lifetime Measurement in Plants^1^

**DOI:** 10.1104/pp.19.00051

**Published:** 2019-03-14

**Authors:** Hongtao Zhang, Eric Linster, Lucy Gannon, Wiebke Leemhuis, Chelsea A. Rundle, Frederica L. Theodoulou, Markus Wirtz

**Affiliations:** a Plant Sciences Department, Rothamsted Research, Harpenden AL5 2JQ, United Kingdom; b Centre for Organismal Studies, University of Heidelberg, Heidelberg 69120, Germany

## Abstract

Targeted protein degradation is an important and pervasive regulatory mechanism in plants, required for perception and response to the environment as well as developmental signaling. Despite the significance of this process, relatively few studies have assessed plant protein turnover in a quantitative fashion. Tandem fluorescent protein timers (tFTs) offer a powerful approach for the assessment of in vivo protein turnover in distinct subcellular compartments of single or multiple cells. A tFT is a fusion of two different fluorescent proteins with distinct fluorophore maturation kinetics, which enable protein age to be estimated from the ratio of fluorescence intensities of the two fluorescent proteins. Here, we used short-lived auxin signaling proteins and model N-end rule (N-recognin) pathway reporters to demonstrate the utility of tFTs for studying protein turnover in living plant cells of Arabidopsis (*Arabidopsis thaliana*) and *Nicotiana benthamiana*. We present transient expression of tFTs as an efficient screen for relative protein lifetime, useful for testing the effects of mutations and different genetic backgrounds on protein stability. This work demonstrates the potential for using stably expressed tFTs to study native protein dynamics with high temporal resolution in response to exogenous or endogenous stimuli.

## Abstract (precis)

Tandem fluorescent protein timers enable non-invasive analysis of protein turnover in intact plant cells, allowing tests of how different genetic backgrounds and treatments affect protein stability.

The protein content of cells is not static but is modified constantly by the combined actions of protein synthesis and degradation, a process termed proteostasis. In recent years, protein breakdown has emerged as an important control mechanism that not only underpins the daily requirements of cellular maintenance but also permits responses to environmental stimuli and progression through different developmental stages. In eukaryotes, the primary pathway for targeted protein degradation is the ubiquitin/proteasome system. Around 6% of the proteins encoded by the Arabidopsis (*Arabidopsis thaliana*) genome are dedicated to this process, underlining its importance in plants (Vierstra, 2009). In plants, the ubiquitin/proteasome system has been shown to target intracellular regulators with central roles in hormone signaling, the regulation of chromatin structure and transcription, morphogenesis, the circadian clock, responses to the environment, self-recognition, and defense against pathogens (Vierstra, 2009; Miricescu et al., 2018). Similarly, the broad importance of proteostasis in humans is exemplified by disease states associated with its dysregulation (Ciechanover, 2013). Proteasome-mediated protein degradation is complemented by autophagy and compartment-specific AAA+ proteases (Nelson et al., 2014; Nelson and Millar, 2015).

Much effort has been expended on analyzing protein synthesis, particularly the role of transcription, which is readily quantified on a genome-wide scale using microarrays and RNA sequencing. These techniques can also be used to identify RNAs that are actively translated via isolation of RNA from polysomes (Zanetti et al., 2005). However, it is generally accepted that the correlation between protein and mRNA abundance is imperfect (Schwanhäusser et al., 2011). Whereas dramatic improvements to proteomics technology mean it is now feasible to measure abundance changes in several thousands of proteins in a single experiment (Bensimon et al., 2012), measuring protein abundance in different genotypes or in response to a stimulus does not distinguish between protein synthesis and degradation. Therefore, specific approaches are required to quantify protein degradation.

Classically, protein turnover has been analyzed using pulse-chase metabolic labeling followed by immunoprecipitation. Protein lifetimes have also been estimated using transgenic plants expressing luciferase reporter fusions in cycloheximide-chase assays (Worley et al., 2000; Dreher et al., 2006). More recently, high-accuracy mass spectrometry has been combined with metabolic labeling to determine the degradation kinetics of many proteins in parallel, with single reaction monitoring mass spectrometry offering improved sensitivity and selectivity for selected proteins of interest (Claydon and Beynon, 2012; Holman et al., 2016). Protein turnover can also be analyzed systematically using pulsed or dynamic stable isotope labeling with amino acids in culture, although such methods are restricted to cell or tissue cultures (Doherty et al., 2009; Schwanhäusser et al., 2011; Boisvert et al., 2012; Welle et al., 2016; Savitski et al., 2018). Although powerful, proteomics approaches lack cellular and subcellular resolution, are biased toward relatively abundant proteins, and the kinetics of metabolic labeling dictate a lower limit for the half-lives that can be quantified (Nelson et al., 2014; Li et al., 2017). This effectively excludes many interesting proteins involved in signaling, such as transcriptional regulators, which are typically of low abundance, may have high turnover rates, and are often restricted to specific cell types. Moreover, a key feature of signaling pathways that cannot readily be captured in a proteomics workflow or an immunoprecipitation experiment is the movement of proteins between subcellular compartments. Intracellular trafficking and turnover of regulatory proteins such as transcription factors, transporters, and E3 ligases play important roles in plant signaling and development (Friml, 2010; Gallagher et al., 2014; Habets and Offringa, 2014; Podolec and Ulm, 2018). In plants, intercellular movement of proteins and peptides is also important in development, for example in root differentiation, where key transcription factors traffic between different cell types (Lee et al., 2006; Long et al., 2015).

Fluorescent proteins (FPs) offer alternative means to study the dynamics of protein turnover with the additional benefit of spatial resolution and potentially high sensitivity. A number of approaches are possible. Photoswitchable FPs may be employed in microscopy-based pulse-chase experiments, whereby a pulse of local light irradiation generates an activated population of a protein species that can be followed in time and space. Alternatively, in a bleach-chase experiment, the protein removal rate can be determined after bleaching a subset of the fluorescently labeled protein population in a given cell (Eden et al., 2011). Both these types of experiments require a time course and fitting models to signal intensities in order to yield turnover estimates. In contrast, fluorescent timers change color as a function of protein age, due to fluorophore maturation. A tandem fluorescent timer (tFT) is a fusion of two single-color FPs that undergo fluorophore maturation with different kinetics, an arrangement that overcomes many of the disadvantages of early single FP timers (Khmelinskii and Knop, 2014; Khmelinskii et al., 2016). The ratio of slow-maturing FP to fast-maturing FP fluorescence intensities provides a measure of protein age through single time point imaging.

The tFT technology was originally developed in yeast (Khmelinskii and Knop, 2014; Khmelinskii et al., 2016) and has been used in animal systems (Donà et al., 2013; Barry et al., 2016; Alber et al., 2018; Durrieu et al., 2018), but despite its broad applicability, it has not yet been applied in plants. Studies combining stable isotope labeling with mass spectrometry indicate that the degradation rates of plant mitochondrial proteins span more than a 50-fold range (Nelson et al., 2013), and a 100-fold range was reported for plastid proteins (Nelson et al., 2014); therefore, different approaches will ultimately be required to cover the entire temporal range of plant protein degradation. The maturation rates of tFT fluorophores can be tuned to be appropriate for the half-lives of the proteins under investigation (Khmelinskii and Knop, 2014). Here, we focus on short-lived proteins, employing a timer composed of the fast-maturing monomeric superfolder green fluorescent protein (sfGFP) and the slower-maturing monomeric red fluorescent protein mCherry that was pioneered for use in yeast (Khmelinskii et al., 2016). We demonstrate that mCherry-sfGFP faithfully reports relative protein lifetime in plants using two exemplars: the N-end rule pathway and auxin signaling proteins. We show that transient expression of tFTs provides a rapid, straightforward method to compare the effects of mutations and different genetic backgrounds on protein stability and that the generation of stable lines expressing tFTs permits analysis of protein stability at subcellular resolution, in different cell types, and in response to exogenous and endogenous stimuli.

## RESULTS

### Proof of Concept: Establishing the Measurement of Relative Protein Lifetime Using Model N-End Rule Reporters

**Figure 1.**
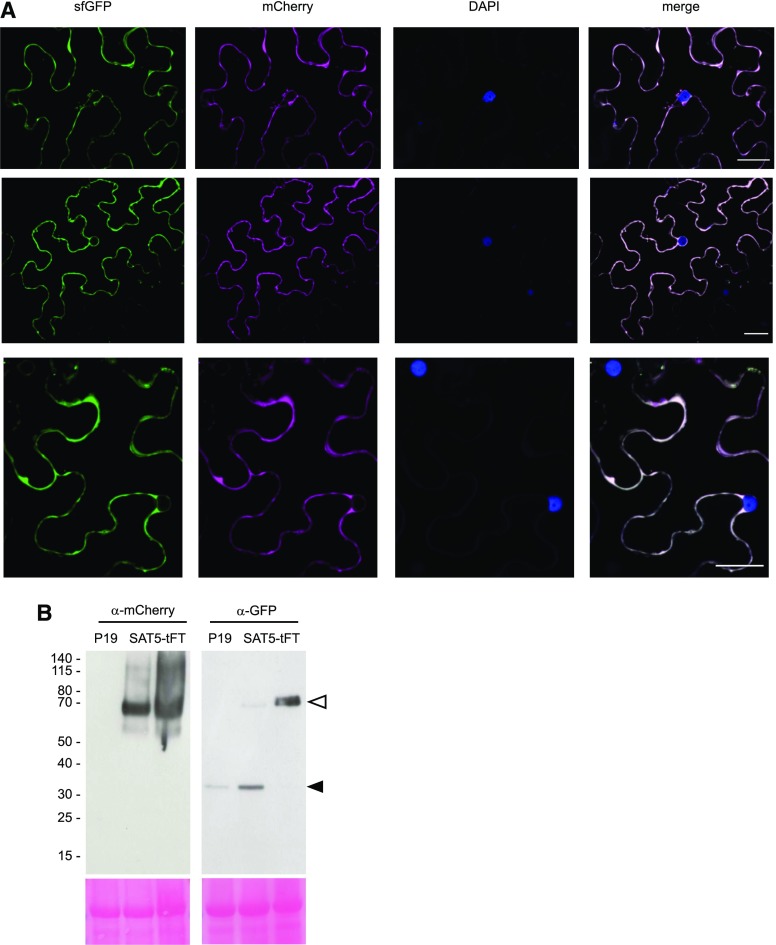
C-terminal mCherry-sfGFP demonstrates cytosolic localization of SAT5. A, *N. benthamiana* leaves were transiently transformed with a construct expressing SAT5-tFT under the control of the *CaMV35S* promoter. The images show false-colored confocal micrographs of three different cells, counterstained with 4′,6-diamidino-2-phenylindole (DAPI) to mark nuclei. Bars = 30 µm. B, Immunoblots of different leaves infiltrated with the P19 suppressor of gene silencing (left lanes) or SAT5-tFT plus P19 (middle and right lanes). Blots (50 mg of protein per lane) were probed with antisera toward GFP or mCherry, as indicated. The gels at bottom show Ponceau S staining following transfer, and the positions of molecular mass markers (kD) are indicated to the left. The white arrowhead indicates the position of SAT5-tFT, and the black arrowhead indicates a P19-specific band.

The range of protein ages that can be interrogated with a tFT depends upon the maturation kinetics of the respective FPs (Khmelinskii et al., 2012). Arabidopsis protein half-lives span a few minutes (e.g. auxin signaling proteins; Worley et al., 2000; Ouellet et al., 2001; Dreher et al., 2006) to many hours or even days (e.g. histones; Nelson et al., 2013, 2014). Whereas sfGFP matures with a half-time of ∼6 min, mCherry maturation can be described by a two-step process with maturation half-times of ∼17 min (first step) and ∼30 min (second step; Khmelinskii et al., 2012). This makes the mCherry-sfGFP timer suitable to study the degradation of proteins with half-lives between ∼10 min and ∼8 h (Khmelinskii et al., 2012). First, we tested the integrity of the mCherry-sfGFP reporter in planta by fusing it to the C terminus of the cytosolic, hexameric protein, SERINE ACETYLTRANSFERASE5 (SAT5; Wirtz et al., 2010), and transfecting *Nicotiana benthamiana* leaf epidermal cells. Free GFP and fluorescent protein dimers (e.g. mCherry-sfGFP) are well known to translocate into the nucleus (Seibel et al., 2007); however, SAT5-tFT was localized in the cytosol and clearly excluded from the nucleus (Fig. 1A). Immunoblotting indicated that the fusion protein was largely intact (Fig. 1B). Together, these results indicate that neither mCherry nor sfGFP was released from the fusion protein in vivo and that the tFT fusion faithfully reported the subcellular localization of SAT5. We cannot fully exclude the possibility that an extremely short-lived sfGFP or mCherry-sfGFP fragment has been released from SAT5-tFT and is diluted in the nucleus to undetectable levels. However, such a theoretical and highly unstable fragment would not interfere, in practice, with the lifetime measurement in planta.

**Figure 2.**
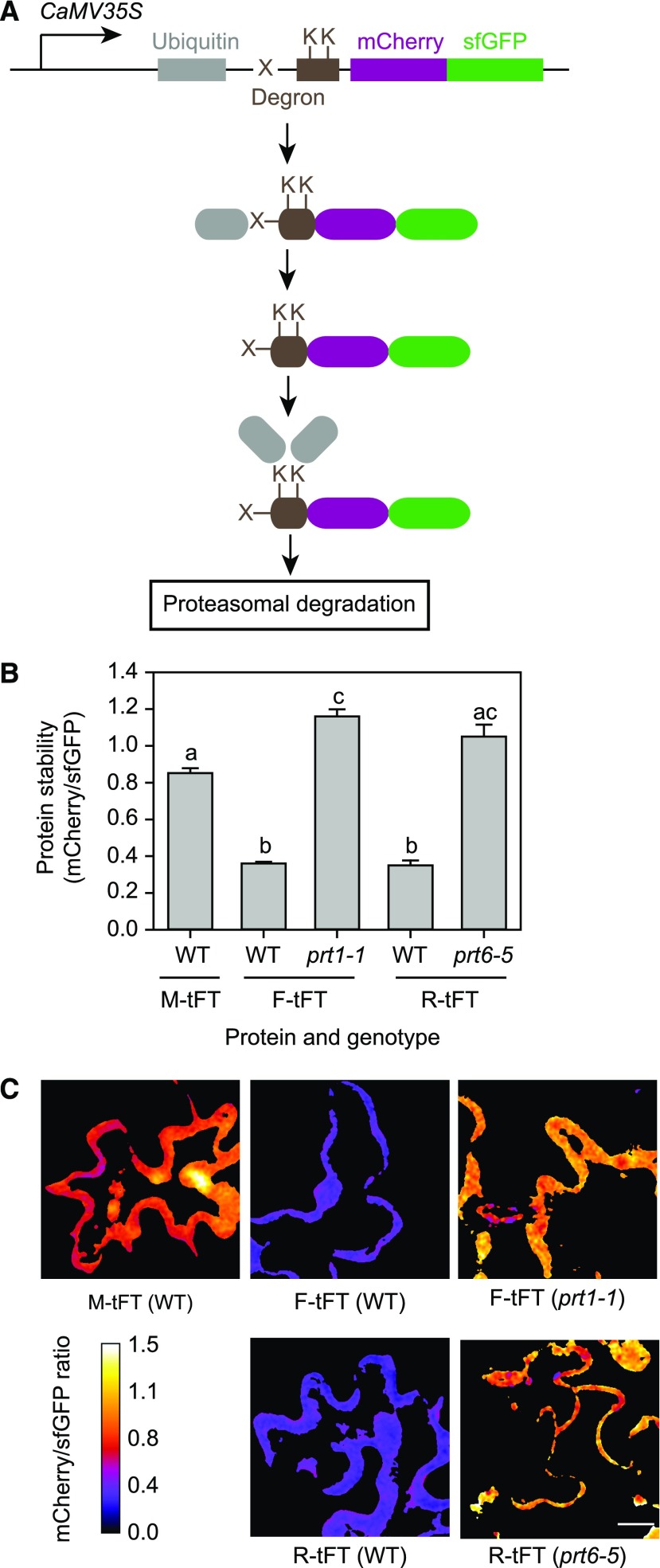
Transient expression of N-end rule tFT reporters in Arabidopsis. A, Generation of N-end rule tFT reporters. Constructs are driven by the constitutive *CaMV35S* promoter and encode a fusion of ubiquitin (gray) to the tandem timer (magenta and green). Deubiquitinating enzymes remove ubiquitin cotranslationally to reveal a new N terminus (a variable residue, indicated by X). A flexible linker (brown box) contains Lys (K) residues, which can be covalently linked to ubiquitin via the sequential activity of E1, E2, and E3 enzymes. Where X is a destabilizing residue (R or F), the respective E3 ligase (PRT6 or PRT1) directs the fusion protein for proteasomal degradation (modified from Khmelinskii et al., 2012). B, Quantification of relative protein stability (mCherry-sfGFP ratio) of the N-end rule tFT reporters in epidermal cells of 5-week-old wild-type (WT) plants and N-end rule E3 ligase-deficient mutants (*prt1-1* and *prt6-5*). Different letters indicate statistically significant differences between groups determined with the one-way repeated measures (RM) ANOVA test (*P* < 0.05, *n* = 4–13). Values represent means ± se. C, Representative false-color images of Arabidopsis leaf epidermal cells expressing N-end rule tFT reporters for calculation of mCherry-sfGFP ratios (blue = unstable; white = stable). The heat map indicates the intensity ratio of mCherry to sfGFP. Bar = 20 µm.

We next designed a Gateway-based cloning scheme to generate plant transformation constructs for expression of proteins of interest fused at the C terminus to the mCherry-sfGFP tFT under the control of a promoter of choice. We created protein timers designed to be targeted for proteasomal turnover according to the N-end rule (N-recognin) pathway for protein degradation (Fig. 2A). The N-end rule relates the half-life of a protein to its N-terminal residue (Bachmair et al., 1986). Substrates for this pathway are generated posttranslationally by nonprocessive endopeptidase cleavage to reveal a new N-terminal amino acid residue or by a combination of cotranslational and posttranslational modifications of the N terminus (Gibbs et al., 2011, 2014). Proteins bearing basic, bulky, or hydrophobic N-terminal residues (classified as destabilizing) are recognized by E3 ligases with different specificities and targeted for degradation by the proteasome (Potuschak et al., 1998; Varshavsky, 2011; Gibbs et al., 2014). In Arabidopsis, PROTEOLYSIS1 (PRT1) is an E3 ligase with specificity for aromatic amino acids, whereas PRT6 targets basic N-terminal residues (Stary et al., 2003; Garzón et al., 2007; Mot et al., 2018). N-end rule substrates can also be created artificially by the ubiquitin fusion technique, in which a genetically encoded N-terminal ubiquitin domain is cleaved in vivo by deubiquitinating enzymes to reveal a destabilizing residue at the N terminus (the so-called N-degron; Varshavsky, 2000; Fig. 2A). Timer constructs thus designed to release Arg-tFT (R-tFT) and Phe-tFT (F-tFT) in planta were transiently introduced into wild-type Arabidopsis (Columbia-0 [Col-0]) and also the *prt6-5* and *prt1-1* mutants, which lack E3 ligases specific for basic and aromatic N termini, respectively (Garzón et al., 2007; Graciet et al., 2009). Met-tFT (M-tFT), which is not a substrate for PRT6 or PRT1, was used as a control. The X-tFT constructs were detected in the nucleus and cytosol (Supplemental Fig. S1). F-tFT and R-tFT were relatively unstable in wild-type cells, with mCherry-sfGFP ratios of 0.35 ± 0.027 and 0.36 ± 0.009, respectively, compared with 0.85 ± 0.027 for M-tFT. In contrast, the stability of the N-end rule reporters was increased significantly in the appropriate E3 ligase mutant background (Fig. 2, B and C). Thus, the mCherry-sfGFP tFT enables quantification of protein stability in a transient expression system. The results also demonstrate that the lifetime of a tFT fusion can be dictated by a single amino acid change (in this case, at the N terminus), indicating that the fate of the mCherry-sfGFP component is influenced predominantly by the degron and not by intrinsic properties of the fluorescent protein fusion itself.

**Figure 3.**
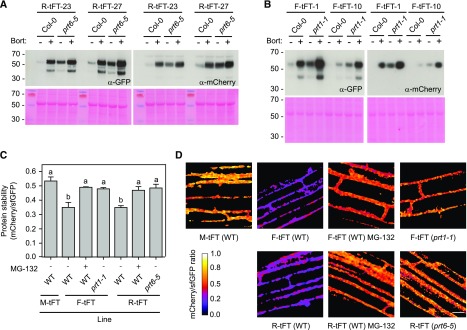
Stable expression of N-end rule tFT reporters in Arabidopsis. A and B, Immunoblots of transgenic lines expressing R-tFT (A) and F-tFT (B). Two independent transgenic lines were selected for each construct and crossed to Col-0 to obtain a control line harboring the same transgene event. Proteins were extracted from 5-d-old seedlings treated with 50 µm Bortezomib (Bort) or dimethyl sulfoxide (DMSO) and subjected to immunoblotting (20 µg of protein per lane) with antisera toward GFP or mCherry. The gels at bottom show Ponceau S staining following transfer, and the positions of molecular mass markers (kD) are indicated to the left. C, Quantification of relative protein stability (mCherry-sfGFP ratio) of the N-end rule tFT reporters in root cells of 7-d-old wild-type (WT) plants and N-end rule E3 ligase-deficient mutants (*prt1-1* and *prt6-5*) treated for 4 h with the proteasome inhibitor MG-132 (50 µm). Different letters indicate statistically significant differences between groups determined with the one-way RM ANOVA test (*P* < 0.05, *n* = 4–7). Values represent means ± se. D, Representative false-color images of Arabidopsis root cells expressing N-end rule tFT reporters for calculation of mCherry-sfGFP ratios (blue = unstable; white = stable). The heat map indicates the intensity ratio of mCherry to sfGFP. Bar = 20 µm.

Transient systems typically afford high, potentially nonphysiological protein expression levels. Therefore, we tested the performance of the X-tFT fusions in transgenic plants. Stable transgenic lines were established in Arabidopsis N-end rule mutant backgrounds and crossed to wild-type (Col-0) plants to generate control lines harboring the same transgene event. Lines were analyzed by immunoblotting: signals corresponding to the expected molecular mass of R-tFT and F-tFT after cleavage of ubiquitin (60.5 kD) were barely detectable in Col-0, but their abundance was increased by treatment with the proteasome inhibitor Bortezomib (Fig. 3, A and B). Abundance of the reporter proteins was considerably higher in the N-end rule mutant backgrounds, *prt6-5* and *prt1-1*, with a modest further stabilization by Bortezomib. A second protein species of approximately 45 kD was detected with the GFP antibody but not the mCherry antibody; this likely represents an mCherry cleavage product known to be formed during cell extract preparation (Gross et al., 2000; Shemiakina et al., 2012). Quantification of mCherry and sfGFP signals by confocal microscopy revealed that F-tFT and R-tFT were less stable than M-tFT in wild-type roots, but the mCherry-sfGFP ratios were increased significantly by the application of a second proteasome inhibitor, MG-132 (Fig. 3, C and D). F-tFT and R-tFT were stabilized in the N-end rule mutants, *prt1-1* and *prt6-5*, respectively, relative to the wild type (Fig. 3, C and D). Taken together, these data indicate that the model N-end rule tFT reporters are turned over by the proteasome in an N-end rule-dependent manner and that this turnover can be faithfully monitored using ratiometric fluorescence measurements.

### Benchmarking tFTs with Auxin Signaling Proteins

We then validated the tFT technique using endogenous proteins whose lifetimes have been measured using established methods. Relatively few studies have systematically addressed protein lifetime measurement in plants, but regulated degradation of Aux/IAA transcriptional regulators mediated by TRANSPORT INHIBITOR RESPONSE1/AUXIN SIGNALING F-BOX (TIR1/AFBs) proteins is well known to play a crucial role in auxin signaling and plant development (Abel et al., 1994; Worley et al., 2000; Gray et al., 2001; Ouellet et al., 2001; Dreher et al., 2006). Aux/IAA proteins form part of the early response to auxin and many are extremely short lived, which enables silencing of the auxin signal once the primary response has been initiated (Abel et al., 1994). This protein family is ideal for challenging the tFT system, since degrons have been identified, different members of the Aux/IAA family vary in their degradation rates, and degradation is influenced by exogenous application of auxin (Dreher et al., 2006). Domain II of Aux/IAA proteins physically interacts with TIR1/AFB and is required not only for rapid degradation but also the auxin-mediated acceleration of degradation (Gray et al., 2001; Ramos et al., 2001; Dharmasiri et al., 2005a, 2005b; Kepinski and Leyser, 2005; Dreher et al., 2006).

**Figure 4.**
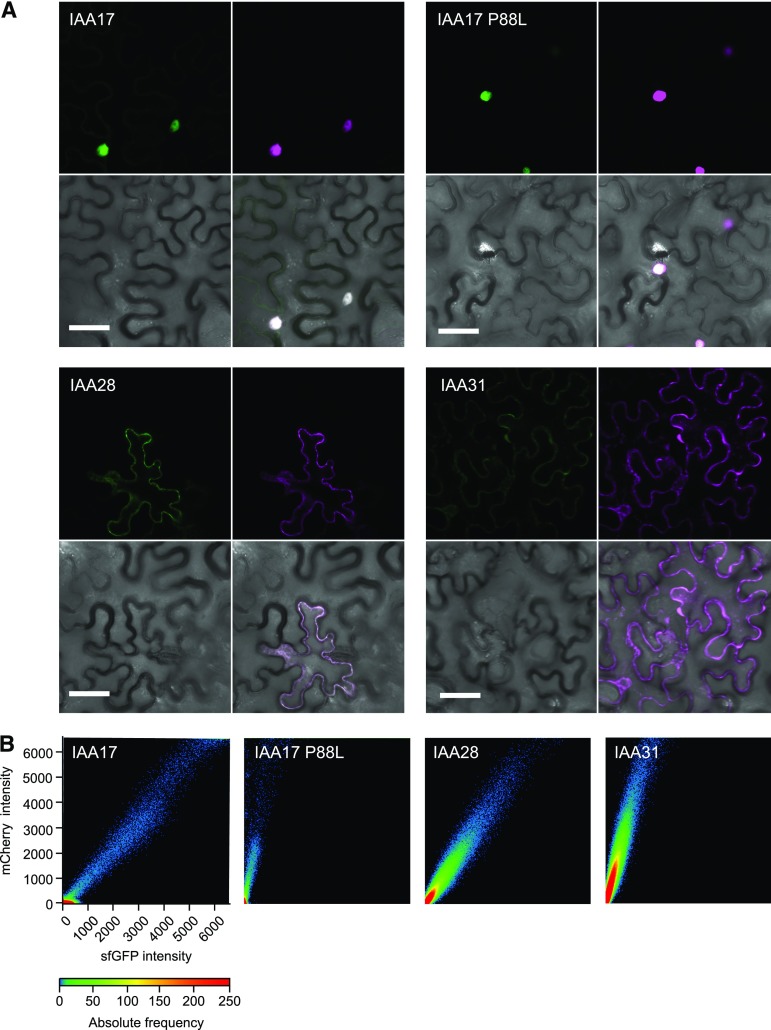
Transient expression of IAA-tFT reporters in *N. benthamiana* epidermis. A, Representative confocal micrographs of *N. benthamiana* leaf epidermal cells agroinfiltrated with different IAA-tFT constructs. Images clockwise from top left: sfGFP, mCherry, merge, bright field. Bars = 50 µm. B, Plots showing relative intensity of mCherry and sfGFP signals on a pixel-by-pixel basis. The color in the plot represents the number of pixels (absolute frequency) that are plotted in that region.

To benchmark the mCherry-sfGFP tFT, we tested three Arabidopsis Aux/IAA proteins with half-lives ranging from a few minutes to hours (Table 1). Initially, we employed transient expression in *N. benthamiana* leaf epidermis, a commonly used system for analyzing fluorescent protein fusions, to compare the lifetimes of IAA17-, IAA28-, and IAA31-tFT fusions. Comparing the intensity of the mCherry and sfGFP signals in agroinfiltrated *N. benthamiana* leaves enabled a straightforward ranking of protein lifetimes (indicated by the slopes of the scatterplots), with the rank order IAA17 ≤ IAA28 < IAA31, consistent with the literature (Fig. 4; Table 1). Introduction of the P88L mutation, which recapitulates a domain II lesion in the Arabidopsis *axr3-1* auxin signaling mutant (Rouse et al., 1998; Ouellet et al., 2001), significantly increased the stability of IAA17-tFT (Fig. 4B). This result demonstrates the utility of transient expression of mutant or truncated proteins in *N. benthamiana* for the rapid analysis of degrons and other structural factors that influence turnover.

**Table 1.** Literature values for Aux/IAA half-lives LUC, Luciferase; NLS, nuclear localization signal. Dashes indicate control treatment.

Protein/Construct	Treatment	Assay	Half-Life	Reference
IAA17	–	[^35^S]Met labeling, cycloheximide chase, and immunoprecipitation from Arabidopsis seedlings	∼80 min	Ouellet et al. (2001)
IAA17_P88L_ (*axr3-1*)	–		∼550 min	Ouellet et al. (2001)
YFP-IAA17-NLS	–	Inducible expression and time-lapse flow cytometry in yeast expressing TIR1	∼22 min	Moss et al. (2015)
UBQ::IAA17-LUC	–	Cycloheximide chase and LUC imaging in Arabidopsis seedlings	11 min	Dreher et al. (2006)
UBQ::IAA17_1-111_-LUC-NLS	–		10 min	Dreher et al. (2006)
UBQ::IAA17_1-111_-LUC-NLS	5 µm 2,4-D		4.6 min	Dreher et al. (2006)
UBQ::IAA28-LUC	–		∼60–80 min	Dreher et al. (2006)
UBQ::IAA28-LUC	5 µm 2,4-D		∼15 min	Dreher et al. (2006)
YFP-IAA28-NLS	–	Inducible expression and time-lapse flow cytometry in yeast expressing TIR1	∼25 min	Moss et al. (2015)
35S::IAA31-10xMyc	–	Cycloheximide chase and quantification with anti-Myc antibody	>20 h	Dreher et al. (2006)
35S::IAA31-10xMyc	10 µm 2,4-D		∼4 h	Dreher et al. (2006)

**Figure 5.**
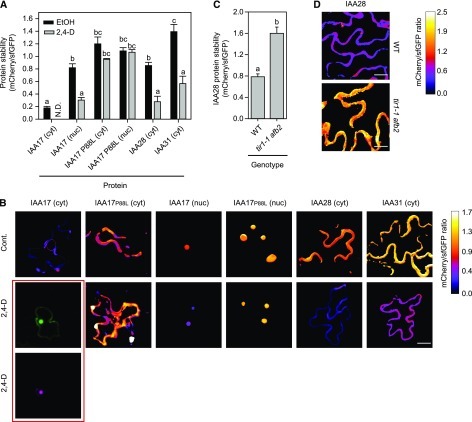
Transient expression of IAA-tFT reporters in Arabidopsis. A, Quantification of relative protein stability (mCherry-sfGFP ratio) of IAA-tFT reporters in epidermal cells of 5-week-old wild-type plants treated for 2 h with 100 µm 2,4-D or ethanol (EtOH; *n* = 4–7). For A and C, different letters indicate statistically significant differences between groups determined with the one-way RM ANOVA test (*P* < 0.05). Values represent means ± se. B, Representative false-color images of Arabidopsis epidermal cells expressing IAA-tFT reporters for calculation of mCherry-sfGFP rations shown in A (blue = unstable; white = stable). The heat map indicates the intensity ratio of mCherry to sfGFP. Nuclear (nuc) and cytosolic (cyt) data were collected separately for IAA17. As a result of significant destabilization of the cytosolic IAA17-tFT by 2,4-D treatment, the mCherry signal was undetectable. In this case, the signals for sfGFP and mCherry of the IAA17-tFT are shown in separate images (red frame). Bar = 15 µm. C, mCherry-sfGFP ratios of IAA28-tFT transiently transformed in the wild type (WT) and the *tir1-1 afb2* double mutant lacking the auxin-activated degradation system (*n* = 8). D, Representative false-color images of Arabidopsis epidermal cells expressing IAA-tFT reporters for calculation of mCherry-sfGFP rations shown in C (blue = unstable; white = stable). The heat map indicates the intensity ratio of mCherry to sfGFP. Bars = 15 µm.

To quantify protein stability and test the effect of exogenous auxin, IAA-tFTs were transiently expressed in Arabidopsis. In agreement with previous studies (Tao et al., 2005; Arase et al., 2012), IAA17-tFT was localized predominantly in the nucleus, with some signal present in the cytosol. Apparently, the cytosolic pool of IAA17-tFT was less stable than nucleus-localized IAA17-tFT (Fig. 5A). Treatment with the synthetic auxin 2,4-dichlorophenoxy acetic acid (2,4-D) reduced the mCherry-sfGFP ratio of nuclear IAA17-tFT from 0.819 ± 0.063 to 0.302 ± 0.042 and reduced the cytosolic mCherry signal to below the limit of detection. As in *N. benthamiana*, the lifetime of IAA17_P88L_-tFT was considerably longer than that of IAA17-tFT in Arabidopsis and was not significantly influenced by auxin treatment (Fig. 5, A and B). In Arabidopsis leaf epidermal cells, the stability of IAA28-tFT was similar to that of IAA17-tFT, whereas IAA31-tFT was longer lived. Auxin treatment reduced the lifetimes of both proteins (Fig. 5, A and B), and removal of auxin receptor function in the *tir1-1 afb2* mutant (Dharmasiri et al., 2005b) resulted in the stabilization of IAA28-tFT (Fig. 5, C and D). This indicates that turnover of the protein is dependent on canonical auxin signaling.

### IAA-tFT Stable Lines Report Auxin Dynamics

**Figure 6.**
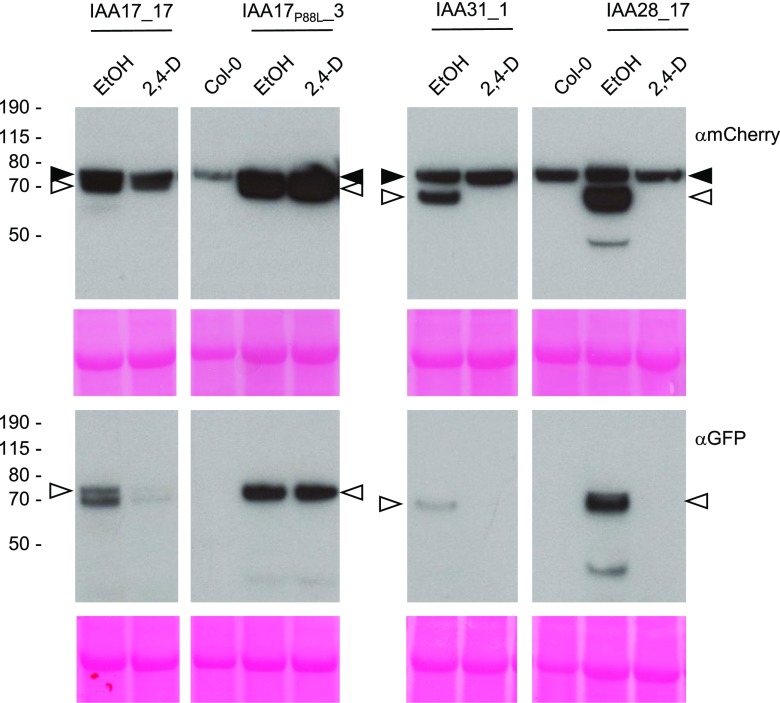
Stable expression of IAA-tFT reporters in Arabidopsis. Immunoblots are from stable Arabidopsis transgenic lines expressing different IAA genes fused to tFT. Five-day-old seedlings were treated with 5 µm 2,4-D or ethanol (EtOH) for 4 h. Blots (40 µg of protein per lane) were probed with anti-GFP or anti-mCherry antibodies (black arrowheads indicate a nonspecific band recognized by the mCherry antibody; white arrowheads indicate the IAA-tFT proteins). The panels at bottom show Ponceau S staining following transfer, and the positions of molecular mass markers (kD) are indicated to the left.

IAA17 and IAA28 play important roles in root growth and development, where their abundance is regulated by auxin (Leyser et al., 1996; Worley et al., 2000; Gray et al., 2001; Rogg et al., 2001; De Rybel et al., 2010; Sato et al., 2015). To explore the behavior of IAA-tFT fusions in roots, Arabidopsis stable transgenic lines were established. Seedlings of lines expressing IAA17-tFT and IAA28-tFT under the control of the strong, semiconstitutive *CaMV35S* promoter were morphologically similar to wild-type plants. However, IAA17_P88L_-tFT roots exhibited an agravitropic phenotype (Supplemental Fig. S2), in agreement with the report of Swarup et al. (2005) that ectopic expression of IAA17_P88L_ (the *axr3-1* gain-of-function mutant) in root epidermis blocked gravitropism. Two of the highest expressing IAA31-tFT lines had significantly fewer lateral roots than the wild-type seedlings (Supplemental Fig. S3), consistent with the phenotype of plants overexpressing the untagged protein (Sato and Yamamoto, 2008). Collectively, these results demonstrate that the consequences of IAA-tFT fusion protein expression are similar to those of ectopically expressing untagged IAA proteins. Immunoblot analysis revealed that the IAA-tFT fusion proteins were largely intact (Fig. 6); therefore, we tested the effect of manipulating auxin levels on IAA-tFT stability. As judged by immunoblotting, application of 2,4-D to seedlings reduced the abundance of IAA17-tFT, IAA28-tFT, and IAA31-tFT but not IAA17_P88L_-tFT (Fig. 6).

**Figure 7.**
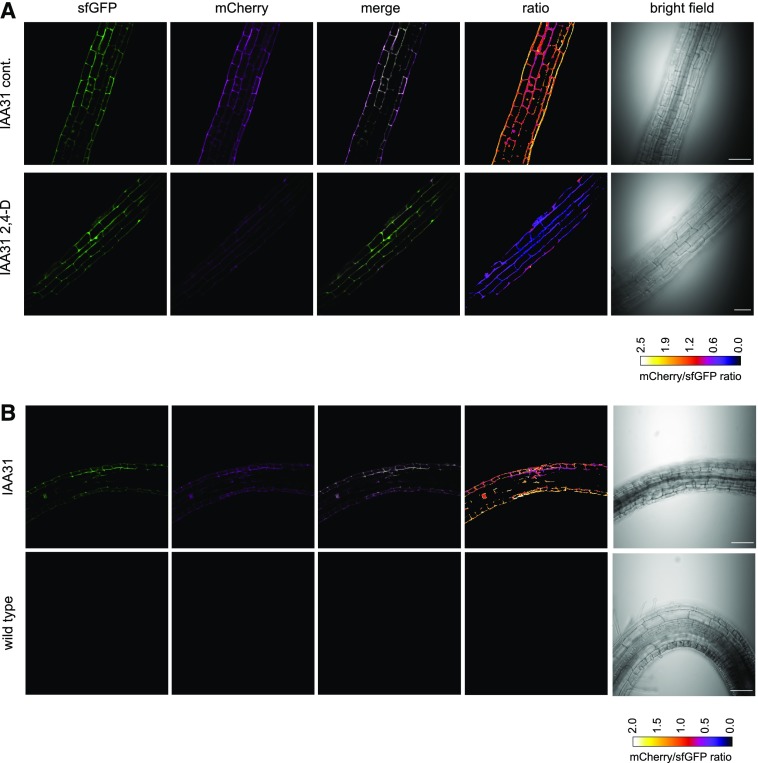
Auxin destabilizes IAA31-tFT in planta. A, Confocal micrographs of primary roots of 6-d-old IAA31-tFT seedlings, treated with 5 µm 2,4-D or ethanol (cont.) for 4 h. B, Confocal micrographs of 14-d-old IAA31-tFT roots turned by 180° for 16 h to induce root bending. Bars = 50 µm.

Next, seedlings were examined by confocal microscopy to assess protein stability in different regions of the root. It was difficult to detect sfGFP or mCherry signals in IAA17-tFT lines under control conditions, but IAA17_P88L_-tFT gave a strong nuclear signal that was evident throughout primary root tips and the differentiation zone (Supplemental Fig. S4). Similarly, IAA28-tFT was difficult to detect in control conditions, although Bortezomib treatment resulted in stabilization (Supplemental Fig. S5). In contrast, IAA31-tFT was detected in the nucleus and cytoplasm of primary root tips (Supplemental Fig. S4A), with the stability apparently reflecting the prevailing auxin gradient (Supplemental Fig. S4B). IAA31-tFT was also stabilized by Bortezomib treatment (Supplemental Fig. S5). Application of 2,4-D to roots grown on vertical plates shortened the lifetime of IAA31-tFT (Fig. 7A). During gravitropism-induced root curvature, auxin accumulates asymmetrically, dependent on the root zone undergoing curvature (Ottenschläger et al., 2003; Swarup et al., 2005; Laskowski et al., 2008; Brunoud et al., 2012). We hypothesized that, in the differentiation zone, the auxin minimum on the underside of a root undergoing gravitropism would lead to stabilization of IAA proteins and the auxin accumulation on the upper side of the root bend would reduce the lifetime of IAA-tFT (Laskowski et al., 2008). Accordingly, rotation of seedlings to induce bending resulted in an increase in the mCherry-sfGFP ratio of IAA31-tFT on the inside of the root bend and a decrease on the outside (Fig. 7B). As expected, the lifetime of the auxin-nonresponsive IAA17_P88L_-tFT did not differ at the site of natural root bends (Supplemental Fig. S4C). Thus, the IAA-tFT transgenic lines demonstrate that the mCherry-sfGFP fusion accurately reports the effects of endogenous and exogenous signals on protein turnover in vivo.

## DISCUSSION

### The mCherry-sfGFP Timer Is Degraded by the Proteasome and Reports Plant Protein Dynamics

Given the importance of proteostasis in plant physiology, accessible methods to monitor and quantify protein turnover in time and space are of great utility, particularly for short-lived proteins involved in signaling. Classical methods for measuring turnover of a specific protein of interest are technically challenging, in part due to the requirement to treat tissue with the global protein synthesis inhibitor cycloheximide, which not only constitutes a severe stress but is also difficult to apply in conjunction with a given stimulus and may not penetrate tissues in a uniform manner. tFTs offer an alternative, noninvasive approach to assess protein turnover in real time in vivo. In this study, we employed model N-end rule reporters and endogenous Aux/IAA proteins to test the suitability of FTs for reporting relative protein lifetime in plants.

The synthetic reporters R-tFT and F-tFT were released from the respective ubiquitin-X-mCherry-sfGFP fusions and, as predicted, were subject to N-end rule-mediated proteasomal degradation in both transient and stable systems. Similarly, the IAA-tFT fusions exhibited a range of lifetimes and were stabilized by proteasome inhibitors. Both the artificial (X-tFT) and native (IAA-tFT) fusion proteins were longer lived in transiently transformed Arabidopsis than in the stable lines, potentially reflecting the higher expression levels typically achieved in transient expression systems, which may overload the proteasome. However, the relative stability of the test proteins in different genetic backgrounds was the same for transient and stable systems. Although strongly influenced by structural features, the half-life of a protein is not an intrinsically fixed property but depends on the protein’s environment (Nelson et al., 2014; Nelson and Millar, 2015). For example, IAA turnover has been investigated by three different methods: immunoprecipitation from [^35^S]Met-labeled seedlings, constitutive expression of luciferase fusions in stable Arabidopsis transgenic plants, and a synthetic degradation assay in yeast (Ouellet et al., 2001; Dreher et al., 2006; Moss et al., 2015; Table 1). The reported half-lives vary 10-fold for IAA17 and ∼2-fold for IAA28, suggesting that this parameter may be influenced by ectopic expression. In this proof-of-concept study, we employed a strong constitutive promoter to facilitate the development of imaging parameters, but in the future it will be instructive to generate stable lines expressing the protein of interest-tFT fusions under the control of native promoters to analyze protein degradation in the most relevant physiological context.

Incomplete proteasomal degradation has been reported for GFP-containing tandem timer combinations in yeast (Khmelinskii et al., 2016), but this was not observed in stable Arabidopsis lines expressing ubiquitin-X-tFT fusions or IAA-tFT fusions. This may reflect differences in proteasome processivity between species (Kraut et al., 2012). An ∼45-kD cleavage product detected by anti-GFP antibodies in extracts from plants expressing the F-tFT and R-tFT constructs was also observed previously in yeast and has been proposed, based on structural considerations, to arise from cleavage of mCherry during sample preparation (Gross et al., 2000; Khmelinskii et al., 2016). Importantly, the exclusion of SAT5-tFT from the nucleus provides strong evidence that GFP is not cleaved from the mCherry-sfGFP tFT in planta, indicating that this tandem timer combination is a robust tool for assessing protein lifetime in plants.

### The N Terminus Is a Determinant of Protein Stability in Plants

Our results provide direct evidence for the relevance of the free N-terminal amino acid residue for plant protein stability, demonstrating that this feature can be recognized by distinct E3 ligases (Varshavsky, 2011; Gibbs et al., 2016). Whereas several studies have shown that the abundance of both artificial and selected endogenous proteins is dictated by the N-end rule in plants (Potuschak et al., 1998; Garzón et al., 2007; Gibbs et al., 2011; Licausi et al., 2011), tFTs show directly that protein turnover is influenced by the N terminus, complementing lifetime measurements performed using tFT or classical cycloheximide-chase methods in yeast and human systems (Varshavsky, 2011; Khmelinskii et al., 2012). The potential importance of the N-terminal amino acid for protein stability has also been shown in the context of the acetylation/N-end rule branch in yeast and plants for a limited number of proteins (Hwang et al., 2010; Xu et al., 2015; Linster and Wirtz, 2018), although the pathway is more nuanced and complex than originally envisaged in yeast (Kats et al., 2018). N-terminal modification of plant proteins is a dynamic and stress-induced process that, in turn, controls environmental stress responses via modulation of protein stabilities (Linster et al., 2015; Vicente et al., 2017). The combination of the ubiquitin fusion technique with the tFT sensor to release proteins with a defined N terminus enables testing the importance of selective translation initiation or N-terminal modifications on turnover of the candidate protein in a fast and cost-effective manner and also provides information on the subcellular localization of the protein species. Furthermore, commercially available selective affinity matrices for GFP and red fluorescent protein can be combined with the tFT tag in a dual-affinity pull-down approach to directly link the relevance of posttranslational modifications to an in vivo-determined turnover rate for a candidate protein.

### IAA-tFT Fusions as an Exemplar for Studying Protein Lifetime in Vivo

Following the positive results with artificial N-end rule reporters, we tested whether the stability of endogenous Aux/IAA proteins could also be determined using tFTs. Auxins act as an adaptor for binding Aux/IAA proteins to the F-box proteins TIR1 and AFB1 to AFB5, which are components of SCF^TIR1/AFB^ ubiquitination E3 complexes (Gray et al., 2001). Formation of the SCF^TIR1/AFB^ complex leads to polyubiquitination and degradation of Aux/IAA proteins (Calderón Villalobos et al., 2012). Accordingly, degradation of IAA28-tFT was inhibited in the *tir1-1 afb2* mutant, which markedly impairs but does not eliminate the auxin response (Dharmasiri et al., 2005b). Domain II of the Aux/IAA proteins contains a well-characterized degron, and the P88L mutation in this motif significantly increased the lifetime of the IAA17-tFT fusion, consistent with pulse-chase studies of ^35^S-labeled IAA17 and the phenotype of the *iaa17*/*axr3-1* mutant (Rouse et al., 1998; Ouellet et al., 2001). Of the three IAA family members tested, IAA31 was the longest lived. Interestingly, domain II is incompletely conserved in IAA31 (Dreher et al., 2006), containing Asp in place of a conserved Gly, comparable to the Gly-to-Glu substitutions found in dominant mutants *shy2-3*/*iaa3* and *iaa18-1* (Tian and Reed, 1999; Reed, 2001). In agreement with the literature, exogenous application of auxin to stably transformed lines increased IAA-tFT turnover, as did manipulation of endogenous auxin by induction of root bending, demonstrating that the system is able to resolve dynamic alterations in protein turnover in vivo. This will offer the possibility to explore microenvironments with locally altered protein stability in future studies. Also, although tFTs were developed as a protein age sensor, the success of the domain II-Venus auxin sensor (Brunoud et al., 2012) suggests that fusion of Aux/IAA proteins or their degrons to mCherry-sfGFP has the potential for the development of second-generation auxin sensors. Finally, tFTs have been used to great effect in yeast-based high-throughput screens to understand mechanisms controlling protein turnover (Khmelinskii et al., 2012; Kats et al., 2018). IAA17_P88L_-tFT provides a proof of concept for extending this approach to plants, for example by high-throughput screening of ethyl methanesulfonate-mutagenized lines expressing a protein of interest fused to a fluorescent timer.

## CONCLUSION

We have demonstrated two complementary tandem timer approaches to study plant proteostasis: a transient expression system for rapid assessment of protein turnover and a stable expression system for analysis of protein stability in near-native contexts. The transient system is straightforward and accessible to any lab with a confocal microscope, requiring only standard filters and laser settings for detection of GFP and red fluorescent protein. Many applications can be envisaged, such as analysis of degrons by mutagenesis and deletion strategies or using loss-of-function lines to test candidate substrates for the ∼1,400 E3 ligases in Arabidopsis, the majority of which have not been characterized (Vierstra, 2009; Hua and Vierstra, 2011). The transient system also provides a rapid means to screen new fluorescent protein combinations to extend the lifetime range of the system or to produce timers with different spectral properties. Stable expression of tFTs offers an even wider range of potential applications. The tFTs are particularly valuable for measuring processes out of steady state, such as signaling events, which are difficult to address using fluorescent switchers (Knop and Edgar, 2014). In contrast to fluorescent switchers, tFTs do not require a physical intervention and can be imaged in single snapshots, which allows analysis of protein turnover with high temporal resolution in a specimen. Consequently, analysis of protein turnover in response to a stimulus such as hormone or light treatment is straightforward, and future development of quantitative imaging protocols will enable even more sophisticated approaches, for example, combining stably expressed tFTs with time-lapse imaging in tractable systems such as the Arabidopsis root (Larrieu et al., 2014) to explore and quantify protein turnover events during growth and development. Although fluorescent timers have generally been used to analyze proteasome-dependent protein turnover, in principle they can be adapted to study autophagy or targeted to organelles such as mitochondria and plastids, which have different degradation machineries (Nelson and Millar, 2015). tFTs have also been developed to study organelle division and partitioning in yeast (Kumar et al., 2018).

In summary, tFTs fill an unmet need for a simple, versatile method to quantify dynamic changes in plant protein stability using live imaging with high spatial and temporal resolution. tFTs provide the basis for the development of a suite of sophisticated tools to study the fascinating and extensive plasticity of the plant proteome.

## MATERIALS AND METHODS

### SAT5 Construct

Full-length SAT5 (AT5G56760) was amplified from Arabidopsis (*Arabidopsis thaliana*) leaf cDNA using the SAT5-tFT_For and SAT5-tFT_Rev primers by PCR and fused with the tFT tag that was PCR amplified from pMaM17 (Khmelinski et al., 2012) using the mCherry_For and sfGFP_Rev primers (Supplemental Table S1). The resulting SAT5-tFT fusion was cloned in the pBinAR vector to allow expression under the control of the *CaMV35S* promoter. Correct integration of SAT5-tFT in the pBinAR-SAT5-tFT vector was verified by sequencing.

### N-End Rule and IAA Reporters

For N-end rule reporters, the Ubi-X-mCherry-sfGFP cassette was amplified from p415-GAL1-Ubi-R-mCherry-sfGFP (pMaM107; Khmelinskii and Knop, 2014) using primers AttB1_ubi-X-tft_For and AttB2_ubi-X-tft_stop and recombined into plasmid pDONR221 to create an entry cassette. F- and M-variants were constructed by site-directed mutagenesis using a QuikChange II XL kit (Agilent Technologies), according to the manufacturer’s instructions. The different entry vectors were then recombined into pB2GW7 (Karimi et al., 2002). For IAA reporters, the mCherry-sfGFP cassette was amplified using primers AttB2r_mCsfGFP_For and AttB3_mCsfGFP_stop and recombined into plasmid pDONRP2R-P3 to create the entry cassette pEN-R2-mCsfGFP-L3. IAA17 (At1g04250), IAA28 (At5g25890), and IAA31 (At3g17600) were amplified from an Arabidopsis root cDNA library by PCR using the primers in Supplemental Table S1 and recombined into plasmid pDONR221 to create entry vectors. IAA17_P88L_ was generated by site-directed mutagenesis using a QuikChange lightning kit. Vectors were sequenced to verify the presence of the mutation and to confirm that no unwanted mutations had been introduced. Each IAA entry vector pEN-L1-IAA-L2 was then recombined with pEN-L4-2-R1,0 (Karimi et al., 2007) and pEN-R2-mCsfGFP-L3 into pB7m34GW,0 (Karimi et al., 2005) to create constructs to express IAA-mCherry-sfGFP under the control of the *CaMV35S* promoter. Stable Arabidopsis transgenic lines were obtained by floral dip (Clough and Bent, 1998). Col-0 was transformed with the IAA-tFT constructs. *prt6-5* (Graciet et al., 2009) and *prt1-1* (Potuschak et al., 1998) were transformed with pHT23 (R-tFT) and pHT25 (F-tFT), respectively. Independent lines were crossed to Col-0 to obtain the same transgene events in the wild-type background.

### Growth of Plants and Treatments

Arabidopsis plants were grown for 5 weeks on soil or 7 d on 0.5× Murashige and Skoog (MS) medium containing 1% (w/v) Suc and 0.6% (w/v) Phytagel under short days (8 h of light) at 22°C during the day and 18°C during the night. For the application of 2,4-D or MG-132 to stably transformed Arabidopsis seedlings, the seedlings were grown for 7 d on 0.5× MS medium solidified with agar and then transferred to 0.5× MS medium supplemented with 5 µm 2,4-D or 50 µm MG-132 for 4 h. 2,4-D and MG-132 were dissolved in 0.1% (v/v) ethanol and 0.1% (v/v) DMSO, respectively, with solvent only used as controls. For the Bortezomib treatment, 5- or 7-d-old seedlings were transferred to 0.5× MS medium supplemented with 50 µm Bortezomib or DMSO for 24 h. Gravity-induced root bending was achieved by 180° rotation of the 14-d-old seedlings stably expressing IAA31-tFT grown on 0.5× MS medium supplemented with 1% (w/v) Suc and 0.6% (w/v) Phytagel. For treatment of transiently transformed Arabidopsis plants, the leaves were detached from intact plants at day 4 after the infiltration with *Agrobacterium tumefaciens* harboring the construct of choice and were fed via the petiole for 2 h with 100 µm 2,4-D dissolved in water. Ethanol dissolved in water served as a control for the petiole feeding.

### Arabidopsis Transformation and Confocal Microscopy

*A. tumefaciens*-mediated transient transformation of 5-week-old soil-grown Arabidopsis plants was performed according to Mangano et al. (2014). The transiently transformed plants were analyzed 4 d after infiltration. Leaves were placed on a water-covered slide and analyzed using a Nikon A1 confocal microscope equipped with gallium arsenide phosphide detectors and solid-state lasers for excitation at 405, 488, and 561 nm. The fluorescence signal was imaged at 525/50 nm after excitation at 488 nm for sfGFP and at 595/50 nm after excitation at 561 nm for mCherry. The laser power of 488- and 561-nm lasers was set to a 1:3 ratio. The spectral properties of mCherry and sfGFP are shown in Supplemental Figure S6. Ratiometric quantification of fluorescence images was performed after applying a Gaussian blur with a σ of 1 and background subtraction in ImageJ (v.1.52h; https://imagej.nih.gov/ij). To produce a ratio image on a pixel-by-pixel basis, signal intensities of the mCherry channel were divided by the intensities of the GFP channel using the image calculator function. mCherry-sfGFP ratios were visualized by changing the grayscale values of the resulting image to false color using the ImageJ lookup table Fire. Since the ratios are sensitive to microscope settings, only ratios calculated with identical configuration of the microscope were compared. For visualization of the nucleus, nuclear DNA was stained by leaf infiltration of 0.3 µm DAPI for 10 min. The DAPI-specific fluorescence was detected at 450 nm after excitation at 405 nm with a Nikon A1R confocal microscope.

### *Nicotiana benthamiana* Transfection and Confocal Microscopy

Growth and agroinfiltration of *N. benthamiana* were performed according to Sparkes et al. (2006) and Li (2011). Images were acquired 2 d after infiltration using a Zeiss LSM 780 confocal microscope with sfGFP excitation at 488 nm and emission at 501 to 522 nm and mCherry excitation at 561 nm and emission at 600 to 622 nm. Within an experiment, all images were acquired using identical settings. The mCherry signal was false-colored magenta for presentation. Scatterplots of mCherry and sfGFP intensities were acquired using the colocalization function of ZEN 2010 imaging software (Zeiss). Colocalization was performed on a pixel-by-pixel basis.

### Immunoblotting

Protein extraction and immunoblotting were performed as described in Zhang et al. (2018). Primary antibodies were used at the following dilutions: anti-mCherry (ab183628; Abcam), 1:3,000; anti-GFP from mouse IgG1κ (clones 7.1 and 13.1; Roche), 1:1,000. The secondary antibodies used were anti-rabbit horseradish peroxidase conjugate (A0545; Sigma) diluted 1:50,000 for mCherry or m-IgGκ BP-HRP (sc-516102; Santa Cruz Biotechnology) diluted 1:5,000 for GFP. Blots were then washed and developed with enhanced chemiluminescence reagent SuperSignal West Pico Chemiluminescent Substrate or SuperSignal West Femto Maximum Sensitivity Substrate (Thermo Fisher Scientific), as required.

### Statistical Analysis

Statistical analysis was routinely performed using SigmaPlot 12.5 software (Systat). Different data sets were analyzed for statistical significance with the one-way RM ANOVA, which uses the Holm-Sidak method for multiple pairwise comparisons. Normality distribution of data points was tested with the Shapiro-Wilk method (*P* to reject was *P* > 0.05). Letters indicate significant differences (*P* < 0.05) in the figures.

### Accession Numbers

Sequence data from this article can be found in the GenBank data library under accession numbers At5g56760 (SAT5), At3g24800 (PRT1), At5g02310 (PRT6), At1g04250 (IAA17), At5g25890 (IAA28), At3g17600 (IAA31), At3g62980 (TIR1), and At3g26810 (AFB2).

### Supplemental Data

The following supplemental materials are available.

**Supplemental Figure S1.** Nuclear-cytosolic localization of X-tFT.
**Supplemental Figure S2.** Phenotypes of seedlings expressing IAA-tFT fusions.
**Supplemental Figure S3.** Phenotypes of seedlings expressing IAA31-tFT.
**Supplemental Figure S4.** IAA-tFT stability in Arabidopsis roots.
**Supplemental Figure S5.** Bortezomib stabilizes IAA28-tFT and IAA31-tFT.
**Supplemental Figure S6.** Attributes of fluorescent reporters used in the mCherry-sfGFP tFT.
**Supplemental Table S1.** Primers used in this study.
